# Optimization and fabrication of a novel 3D-printed variable density range modulation device for proton FLASH beams

**DOI:** 10.1002/mp.70013

**Published:** 2025-10

**Authors:** Wenbo Gu, Khayrullo Shoniyozov, Kai Mei, Alexander Lin, Wei Zou, Lei Dong, Peter B. Noël, Boon-Keng Kevin Teo

**Affiliations:** 1 Department of Radiation Oncology, Hospital of the University of Pennsylvania, Philadelphia, Pennsylvania, USA; 2 Department of Radiology, Hospital of the University of Pennsylvania, Philadelphia, Pennsylvania, USA

## Abstract

**Background:**

For proton FLASH therapy, range-modulating devices are inserted in the beam path to create a spread-out-Bragg-peak (SOBP) for ultrafast delivery using a single energy pencil beam scanning technique. Current design typically consists of uniform density spikes with range modulation achieved by changing the area and height of the spikes, which has limited structural stability and modulation flexibility.

**Purpose:**

We present a new class of 3D-printed range-modulating devices for particle therapy with spatially modulated density.

**Methods:**

PixelPrint technology (Laboratory for Advanced Computed Tomography Imaging, University of Pennsylvania, PA) was used to 3D-print the variable density range-modulator, by continuously varying the ratio of filament to air in each voxel. With specific thickness and spatial density modulation, SOBP of varying widths can be created. A calibration phantom was 3D printed and scanned by a dual-energy computed tomography (CT) scanner to characterize the physical and radiological properties of the PixelPrint technology. We developed an inverse optimization algorithm to generate the density map for producing SOBP from monoenergetic proton beam and verified by MCsquare (http://www.openmcsquare.org/), an open-source Monte Carlo (MC) simulation platform. The range modulation characteristics were measured using a multi-layer ionization chamber (MLIC) under monoenergetic proton field irradiation.

**Results:**

The proposed optimization framework generated the density distributions for multiple SOBP widths. MC simulation verified the width and flatness of created SOBPs. The CT scan of a 3-cm SOBP modulator showed good fidelity of the desired density distribution, except for the highest density regions. MLIC measurements confirmed the accuracy of the produced SOBP with multiple proton beam energies.

**Conclusion:**

A novel variable density range-modulating device for proton therapy was successfully developed. These devices have the potential to be handled easily and significantly speed-up proton therapy treatment delivery.

## 1 | INTRODUCTION

FLASH radiotherapy (RT) utilizing ultra-high dose rates is a rapidly evolving field with the potential to revolutionize cancer treatment. Compared with conventional dose rates used in the clinic, ultra-high dose rate radiation (typically ≥ 40 Gy/s) provides a potential benefit of decreasing normal tissue toxicity while achieving an equipotent tumor-killing effect. The characteristic FLASH effect has been reported in multiple preclinical animal studies using electron,^1–4^ photon^5^ and proton^6^ irradiations. In addition, the short treatment time due to ultra-fast delivery (0.1 s or less) can potentially mitigate uncertainties from intra-fractional motion if administrated precisely, permitting a reduction in motion-associated treatment margin to further spare normal tissue.^7^

Compared with conventional electron or x-ray therapy, proton radiation therapy utilizing high energy charged particles offers an advantage for cancer treatments due to its characteristic depth dose deposition profile known as the Bragg peak (BP). The finite range and a sharp distal dose fall-off at the BP permit lower radiation to normal tissue distal to the cancer target. Additionally, the high ionization density of charged particles at the BP introduces an enhancement of biological effectiveness over photon beams.^8^ Combining FLASH and proton therapy enables more localized dose distribution from the BP and increases relative biological effectiveness to potentially enhance the FLASH effect.^6,9,10^

Pencil beam scanning (PBS) technology has been widely implemented in proton therapy systems to utilize pristine monoenergetic pencil beams with a narrow lateral dose profile. The advantages of the highly conformal dose and intrinsic high scanning dose rate^11^ compared to conventional photon or electron therapy make it well-suited for implementing FLASH RT. However, a challenge of PBS technique is the long energy switching time^11^ required for volumetric irradiation. For example, for a cyclotron-based system, energy selection is accomplished by varying the thickness of energy-degrading material and adjusting beamline electromagnetic currents for the reduced energy. The switching time between energy layers typically ranges from 16 ms to 2 s depending on the facility^12^ and a conventional PBS delivery with multiple energy layers can take from 20 s to 2 min to deliver a treatment field. Although recent advancements have enabled field delivery within approximately 5 s using rapid energy-layer switching techniques,^12^ achieving consistently ultrafast delivery to meet FLASH dose rate specifications remains challenging, particularly when multiple energy layers are required. To achieve fast PBS delivery without the need for layer changes, a hardware-based range modulation device can reduce the treatment time to less than a fraction of a second.

The spread-out-Bragg-peak (SOBP) can be created from a monoenergetic beam by using a passive stationary device, or the range modulator, which can be placed in the beam path.^6,11,13–15^ By placing the peak-and-valley shaped energy-degrading materials along the beam path, the scatter of the particle beam and range straggling will redistribute the beam intensities and energies downstream to create a SOBP, if properly designed. Methods used to construct these range modulating devices have been explored using machining techniques or 3D printing.^15,16^ Current range modulator design typically consists of uniform density spikes that can be manufactured using a traditional 3D printer with fixed density binary printing.^15,16^ The range modulation is achieved by changing the area and height of the spikes. Ridge filters are a commonly employed design in FLASH RT research.^10,15–21^ Because of the process of 3D printing, the height of these spikes is limited by the stability of the structure, as it needs to be self-supporting, therefore constraining the flexibility in design and manufacture. The sub-millimeter precision required at the peak of a spike also imposes challenges to 3D printing technique. In addition, the presence of oblique edges in the beam direction reduces the robustness of the filter performance with respect to transverse position variations.

PixelPrint technology is a novel solution to 3D print CT phantoms with variable densities.^22–24^ By continuously varying the ratio of filament to air in each voxel, it is capable of printing structures with non-uniform densities to sub-millimeter voxel resolution. A lung phantom printed using this technology has demonstrated good fidelity to geometry and attenuation profiles.^22,23^ With these advantages, a new class of range modulation devices can be developed with high modulation flexibilities over conventional 3D printing techniques.^18^ We aim to apply PixelPrint technology to construct range modulators that can achieve better range modulation and easier to handle, particularly for FLASH applications.

## 2 | METHODS

The objective of this work is to design a variable density range modulation device (VDRMD) and demonstrate its feasibility for range modulation in proton therapy. We developed an inverse optimization method to design the density pattern of the devices. A Monte Carlo simulation was used to model the generation of variable SOBP from monoenergetic proton beams and to verify the design prior to 3D printing. A variable density device was printed by PixelPrint (Laboratory for Advanced Computed Tomography Imaging, University of Pennsylvania, PA), and its performance was validated by proton beam measurements with a multi-layer ion chamber (MLIC) (Zebra, IBA Dosimetry, Belgium).

### 2.1 | PixelPrint calibration phantom

When 3D printing a variable density phantom, traditional methods involve segmenting regions of interest to multiple densities, converting those regions to separate triangulated surface geometry models (like STL files), printing them respectively and later assembling them together.^25–27^ These methods are limited by spatial resolution and often result in nonsmooth or unrealistic transitions between regions of different densities. PixelPrint directly translates digital imaging and communications in medicine(DICOM) image slices into G-code, a commonly used machine language defining 3D printing parameters. For each layer of the designed 3D phantom, PixelPrint generates arrays of spaced, parallel filament lines. Although the spacing between lines remains fixed, the widths of the filament lines vary, producing a partial volume effect that results in varying densities in the final CT slice. In high-density areas, the filament lines are thicker, whereas in low-density areas, they are narrower. For example, with spacing at 1.0 mm, lines with widths continuously changing from 1.0 to 0.1 mm correspond to continuously reducing infill ratio from 100% to 10%. The infill ratio represents the proportion of the filament occupying a given unit volume. A more detailed description of PixelPrint is provided in Mei et al.^22,28^ It was designed to produce patient-specific phantoms for CT imaging, offering high spatial resolution, realistic attenuation profiles, and lifelike textures. This work demonstrates the feasibility of range modulation and presents a potential application in proton therapy.

Polylactic acid (PLA) filament (Atomic Filament, Kendallville, IN) was used was used in PixelPrint for this study due to its high reliability and cost-efficiency. In order to utilize PixelPrint for proton therapy, the properties of PLA under different infill ratios were characterized. For this characterization, correspondence between density and relative proton stopping power ratio (SPR) to water was needed. In the Monte Carlo simulation, exact mass density, electron density, and density to Hounsfield unit (HU) conversion were required as input. 3D printing requires the input of infill distribution; therefore, it was essential to quantify the exact density of PLA and convert the designed density to infill ratio. The manufacturer-supplied density of PLA is 1.25 g/cm^3^, with an uncertainty of 5%, but this is not accurate enough for proton radiotherapy applications. Therefore, a calibration phantom was printed to characterize the physical properties of the PLA filament. The calibration phantom is composed of nine cubic segments, each measuring 3 cm × 3 cm × 2 cm, with infill ratios ranging from 20% to 100% in increments of 10%. And the exact density of each section was calculated as the product of the infill ratio and the full density of PLA (*ρ*_full_).

The calibration phantom was scanned using a CT scanner (Definition Edge, Siemens Healthineers, Germany), in dual-energy mode at 80 kVp, followed by 140 kVp. Iterative image reconstruction was used to reduce image noise, and a voxel spacing of 0.2 mm (Qr66 kernel) was used. The same CT protocol was used to scan the printed modulator described in the following sections to maintain consistency with calibration while preserving sufficient spatial density information. The average HU of each section in the calibration phantom was extracted from the CT image and the relative electron density to water (*ρ*_*e,rel*_) and SPR of each infill ratio were calculated from the dual-energy CT data.^29^ The mass densities *ρ* (g/cm^3^) of 90% and 100% infill sections were calculated from relative electron density based on the following equation from AAPM Task Group Report 186^30^:

(1) $$\rho =-0.1746+1.176\rho_{e,rel}\left(R^{2}=0.99992\right),$$

which has a maximum error of less than 1%. Both 90% and 100% infill ratios were used to determine *ρ*_full_ because the full density may not be printed accurately due to the upper infill limit of PixelPrint.

### 2.2 | Density optimization framework

The designs of VDRMDs to produce different SOBPs were generated by an inverse analytical framework to optimize the density distribution. The depth dose of an analytical monoenergetic 226 MeV proton beam was used in the optimization. To utilize the analytical BP in the optimization, the following assumptions similar to Akagi et al.^13^ were made:

1. The monoenergetic proton beams are parallel to each other.
2. The proton fluence does not change with the modulator in the beam path.
3. The shape of the Bragg curve does not vary with its residual range and the lateral position. The scatter effect introduced by the modulator is neglected.

**FIGURE 1:**
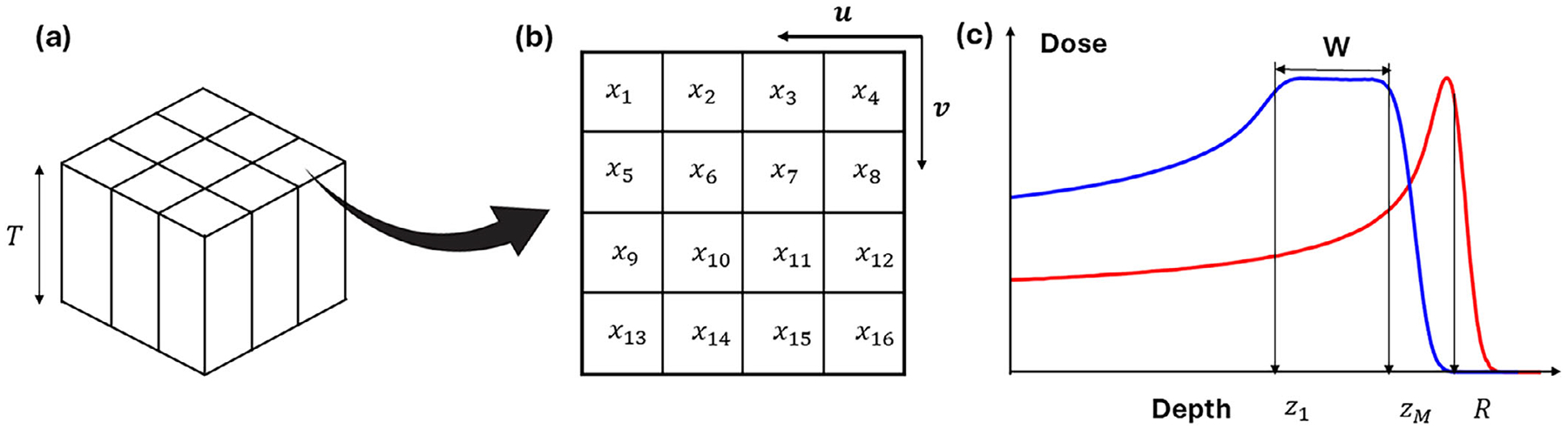
(a) Schematic illustration of the proposed 2D modulated VDRMD with thickness *T*. The device has repeated square density patterns in the lateral plane and constant density along the depth direction. (b) The 2D density map of single segments consists of 16 pixels. *x*_*n*_ represents the density of *n*th pixel. Unit vectors *u* and *v* represent the two lateral directions. (c) The depth dose of a SOBP (blue) modulated from one of the BP curves (red). R is the range of pristine BP and [*z*_1_*, z*_*M*_] is the depth range of SOBP plateau.

The modulator was designed with varying densities in the lateral plane perpendicular to the beam and constant densities along the beam direction. This 2D modulation type was sufficient to generate universal SOBP based on the experience of conventional ridge filters. Additionally, it helped to reduce the degeneracy issue during optimization. To create a universal SOBP, the lateral plane consisted of repeat square density patterns to cover the necessary field size, as shown in Figure 1a. Each pattern was segmented into N square pixels, with densities written as a vector *x* = [*x*_1_*, x*_2_, … .*, x*_*N*_]^*T*^. A schematic illustration of the device with *N* = 16 is shown in Figure 1b.

The mass densities were then converted to relative proton stopping power ratio (SPR) to water (*SPR*(*x*) = *S*(*x*)), for analytical dose calculation. *S*(*x*) was acquired from the calibration phantom described in section II.1. Since SPR is directly correlated with the density of the printing material with one-to-one correspondence, we use the terms SPR and density interchangeably in this work.

Assume the desired range of the SOBP plateau region was discretized into M points of equal step size, as shown in Figure 1c), then the depth of the SOBP region of the modulator can be denoted as *z* = [*z*_1_*, z*_2_, … *, z*_*M*_]^*T*^, and the SOBP width of the device is written as: *W* = *z*_*M*_ − *z*_1_.

The thickness of the device depends on the width of SOBP and the range of printed density. The PLA filament has a full density of around 1.2–1.25 g/cm^3^, and PixelPrint technology can 3D print from 10% to 100% infill ratio. Among them, 0.2–1.1 g/cm^3^ is a range where density can be printed more reliably. Therefore, we set the maximal density (*ρ*_*max*_) of 1.1 g/cm^3^ and a minimal density (*ρ*_*min*_) of 0.2 g/cm^3^. The modulator thickness, denoted as *T*, is calculated from the following equation:

(2) $$S\left(\rho_{\text{max}}\right)*T-S\left(\rho_{\text{min}}\right)*T=W,$$

where *S*(*x*) converts density to relative SPR. Although Equation (2) provides the minimum thickness to achieve the specified density range, using this exact value overly constrains the density modulation. To address this, we increased *T* by a factor of 1.15, corresponding to relaxing both the minimum and maximum density limits by approximately 0.05 g/cm^3^. This provides additional flexibility during optimization, reducing the likelihood of reaching the density bounds prematurely. Although a larger *T* could be used, it would further increase device thickness and, consequently, extend manufacturing times.

It is worth noting that due to the existence of minimal density, the distal end of SOBP will be pulled back proximally from the original BP range by *ρ*_*min*_ * *T*, therefore, the depth of distal end of SOBP is

(3) $$z_{M}=R-S\left(\rho_{\text{min}}\right)*T.$$

where *R* is the beam range in water of the pristine proton beam, defined at distal 90% level. Once the desired width of SOBP is determined, the depth *z* for the SOBP range could be calculated from Equations (2–3).

The dose at depth *z*_*m*_ in water after the modulator is denoted as *D*_*m*_ (*m* = 1, … *, M*). According to the above-mentioned assumptions, the cumulative dose from the sum of shifted monoenergetic depth doses through the variable density modulator can be written as:

(4) $$D_{m}(x)=\sum_{n=1}^{N}B\left(z_{m}+S\left(x_{n}\right)T\right),$$

where *B*(*z*) is the depth dose of the original Bragg peak at depth *z*. It includes the range modulation from density pixels *x*_*n*_ (*n* = 1, … *, N*).

The global optimization function is formulated as follows

(5) $$\underset{x}{\text{argmin}}\sum_{p=1}^{M-1}\sum_{q=p+1}^{M}{\left(D_{q}(x)-D_{p}(x)\right)}^{2}+\gamma \left(∥L^{u}x{∥}_{1}+∥L^{v}x{∥}_{1}\right)\;\text{Subject}\;\text{to}\;\rho_{\text{min}}\leq x\leq \rho_{\text{max}},$$

where *L*^*u*^ and *L*^*v*^ are the derivative matrices for the density map, along the direction *u* and *v* as shown in Figure 1b. *D*_*q*_(*x*) and *D*_*q*_(*x*) represent the dose at depths *z*_*p*_ and *z*_*q*_ within the SOBP (*p* = 1, … *, M* − 1, and *q* = *p* + 1, … *, M*), as defined in Equation (4). For each *p*, with *q* ranging from *p* + 1 to *M*, the term $\sum_{q=p+1}^{M}{\left(D_{q}(x)-D_{p}(x)\right)}^{2}$ quantifies the cumulative squared difference in dose between the current depth *z*_*p*_ and all subsequent depths *z*_*q*_ until the end of the desired SOBP. An ideal, perfectly flat SOBP would yield identical dose values at all depths (*D*_*q*_(*x*) = *D*_*p*_(*x*)∀*p, q*), resulting in a zero contribution from this term. By applying this calculation for all *p*, from 1 to *M* − 1, the first term imposes a penalty whenever there are dose differences between any pair of depths within the SOBP. This encourages uniformity in the dose distribution across the entire SOBP region, thereby promoting a flat SOBP profile.

The second term is the total variation (TV) regularization to the density map, along the two orthogonal directions of the SPR map (*u* and *v* as shown in Figure 1b). It encourages a smooth pixel-wise density distribution in the lateral directions, which reduces potential 3D printing errors. The derivative matrices also include the penalization between the first column/row and last column/row in each pattern, therefore when printing the entire modulator with multiple repetitions of the pattern, the transitions are smoother. γ is the weighting parameter for the total variation regularizations. The function is solved by an in-house developed optimizer using FISTA algorithm,^31^ a fast-iterative shrinkage-thresholding algorithm.

The density distribution generated from the optimization function depends on the initialization density *x*. So, solving the function generates the density values but does not fully optimize the positions of density pixels relative to each other. For example, a random initialization generates randomly distributed densities. Based on the assumptions, scatter is not considered to be impacted by relative density positions, so the dose shift is independent of the density order. The assumptions would break down when excessive heterogeneity is introduced to the proton beam, as proton particles are sensitive to local tissue heterogeneities. In this work, we use a cone-shape distribution for initialization, to mimic the shape of traditional pyramid-like ridge filters, so that the center has a higher density than the peripheral.

### 2.3 | MC simulations

Density distributions of VDRMDs with 1, 2, and 3 cm SOBP modulation were optimized using Equation (5) and cone-shaped initialization. MCsquare,^32^ a fast Monte Carlo (MC) simulation tool for proton dose calculation was used to verify the SOBP plateau width and dose distribution in a water phantom. The lateral dimension of the phantom was set to 6 × 6 cm^2^ with a pixel resolution of 1 mm transverse to the proton beam and 0.5 mm along the beam direction. Square 10 × 10 cm^2^ fields with 5 mm spot spacing for proton beam energies of 150, 180, 210, and 225 MeV were simulated. Each simulation utilized 5 × 10^7^ particles to achieve a statistical uncertainty under 2%. In addition, a 3 cm SOBP with randomly distributed densities was optimized and validated with MC to demonstrate how the spatial arrangement of density affects the resulting SOBP.

For comparison, we also designed a conventional pyramid-shaped ridge filter to achieve a 3 cm SOBP. The weight (cross-sectional area of the step) and thickness (height) of each step in individual ridges were determined using the empirical equations (2–4) described by Jette et al.^33^ Each ridge was modeled with a base area of 5 × 5 mm^2^, 35 steps, and a pixel resolution of 0.5 mm. The resulting ridge filter geometry was then imported into the same Monte Carlo simulation framework used for the variable density devices, to compare the SOBP profiles generated by both approaches.

### 2.4 | SOBP measurements

The density map of the 3 cm cone-shaped SOBP device was then converted to infill ratio and sent to PixelPrint for 3D printing. The devices were printed using fused-filament 3D printer (Lulzbot TAZ 6 with M175 tool head, Fargo Additive Manufacturing Equipment 3D, LLC Fargo, ND, USA) using a 0.25 mm brass nozzle. The printed VDRMD was scanned using the same CT protocol as the calibration phantom and compared against the source file. The depth dose profiles derived from the printed device were measured using the MLIC in a clinical proton pencil beam scanning nozzle of the same field parameters as the MC simulation. The MLIC has a stack of 180 parallel plate ion chambers spaced 2 mm in depth direction with 2.5 cm diameter collecting electrodes.

The shape of the central axis depth dose was compared among analytical calculation, MC, and measurement. The SOBP width was calculated following AAPM TG 224,^34^ which is defined as the distance in water between the proximal and distal 90% dose (d_d_90-d_p_90). It is worth noting that there is a difference of SOBP width definition between optimization and evaluation. In Equations (2–5) the width *W* is the flat region within SOBP, so the first term in Equation (5) penalizes and encourages a flat dose distribution along the width *W*. In the evaluation, the width is d_d_90-d_p_90, since the flat region is not easy to quantify. It is also consistent with the standard definition. Therefore, when we designed a 3 cm SOBP, it is expected that d_d_90-d_p_90 will differ slightly from W.

In addition to depth dose profiles, lateral dose distributions and spot sizes were measured using the Lynx detector (IBA Dosimetry, Belgium), which uses a charge-coupled device (CCD) camera. For lateral dose profile, a 5 × 5 cm^2^ open field of 180 MeV protons was used. 19 cm solid water phantom was placed upstream of the detector to ensure that measurements were obtained within the SOBP plateau, with a 10 cm air gap (AP) maintained between the printed modulator and the solid water surface. Spot sizes, quantified as *σ* in both the *X* and *Y* directions, were measured using a single 180 MeV proton spot without solid water, with air gaps ranging from 5 to 20 cm.

## 3 | RESULTS

### 3.1 | Density and SPR calibration

**FIGURE 2:**
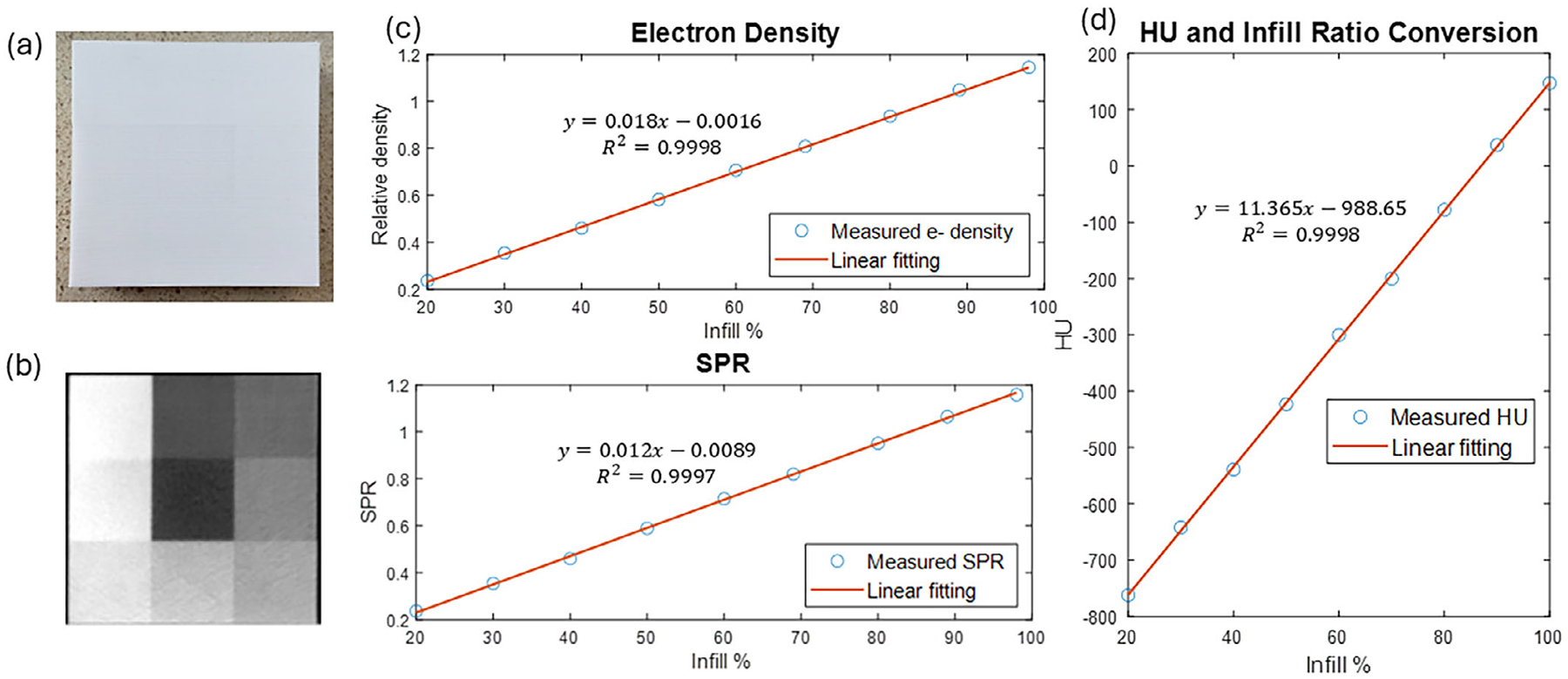
(a) The calibration phantom and (b) the transverse plane of the CT scan. The central block has a 20% infill ratio and the upper left block has a 100% infill ratio. (c) The correspondence of relative electron density and SPR to infill ratio with HU. (d) The HU and infill ratio conversion curve.

The printed calibration phantom and its transverse CT image are shown in Figure 2a,b. From the calibration phantom, electron density and SPR show good linearity with infill ratio (*R*^2^ = 0.9998 and 0.9997, respectively). The conversion between infill ratio and HU are shown in Figure 2d, with *R*^2^ = 0.9998. The highest density PLA section (infill ratio 100%) is shown to have mass density of 1.19 g/cm^3^ and relative electron density to water of 1.1535. Imperfection of printing is observed at the calibration phantom at high infill ratio sections, with intended 90% and 100% infill turning out to be 89% and 98%, respectively. The lower infill ratio sections show difference between design and print of less than 0.5%.

### 3.2 | Optimization and MC simulation

**FIGURE 3:**
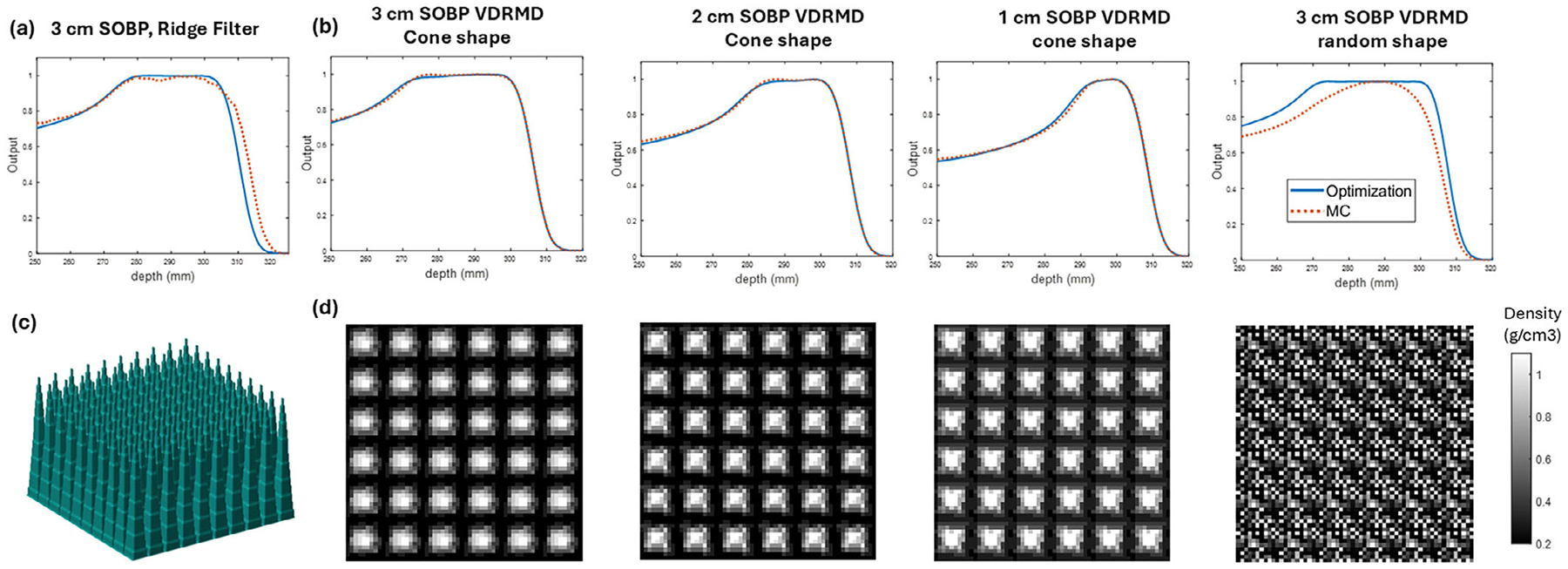
(a) Central axis depth dose in water from a conventional ridge filter designed to achieve a 3 cm SOBP. (b) Central axis depth dose in water from three cone-shaped and one randomly distributed VDRMDs. Solid blue lines represent the analytically optimized dose profiles based on the assumptions in Section II.2, and red dotted lines are the corresponding Monte Carlo simulation results. (c) 3D rendering of the simulated conventional ridge filter. (d) Corresponding density maps for the VDRMDs.

MC simulation of depth doses in water along central axis from a 225 MeV proton beam and the corresponding density maps are shown in Figure 3. The range of MC dose and analytical dose has a few millimeter differences due to a slightly different beam data for 226 MeV used. The depth doses shown in Figure 3 are shifted so the distal edge of MC and optimization align for easier comparison.

With the use of cone-shaped distribution for initialization as described in Section II.2, the optimized density pattern has a high density in the center and lower density in the peripheral, with density ranging from 0.2 to 1.1 g/cm^3^. Each density pattern is 10 × 10 pixels with a 1 mm resolution. Repeating the pattern six times in each transverse direction results in a 6 × 6 cm^2^ size device. The MC simulated depth dose shows similar SOBP profiles with analytical results created from shifted Bragg peaks, affirming the validity of the assumptions made in Section II.2 for computing the density modulation. There is a slight increase in the proximal SOBP in MC simulation compared with optimization input value W, indicating a relative enhancement from high density components in the MC simulation. However, as shown in Figure 3 last column, the perturbations introduced by randomly distributed densities prevent the formation of a stable, flat SOBP.

MC simulations demonstrate that the designed variable density devices can modulate beams and produce a variable width of SOBP from monoenergetic proton beams. The d_d_90-d_p_90 width of the resultant SOBP is listed in Table 1. The MC simulated SOBP width is 0.5–1.3 mm narrower than the optimization, which may be attributable to the effects of scattering that are not considered in the optimization. There is a discrepancy between the designed and evaluated width due to the different definitions of SOBP width used in optimization and evaluation, as described in Section II.3. We produced three devices with d_d_90-d_p_90 widths of 33.1,24.5, and 14.8 mm.

The MC simulation results for the conventional ridge filter with fixed density and variable thickness modulation are also presented in Figure 3. Compared to the cone-shaped VDRMD, the conventional ridge filter exhibited larger deviations between Monte Carlo and analytical optimization results. In addition, the depth dose profile from the ridge filter showed reduced dose homogeneity within the SOBP and a less sharp distal fall-off, indicating the improved modulation performance and scatter control of the proposed variable density design.

### 3.3 | 3D-printed VDRMD

**FIGURE 4:**
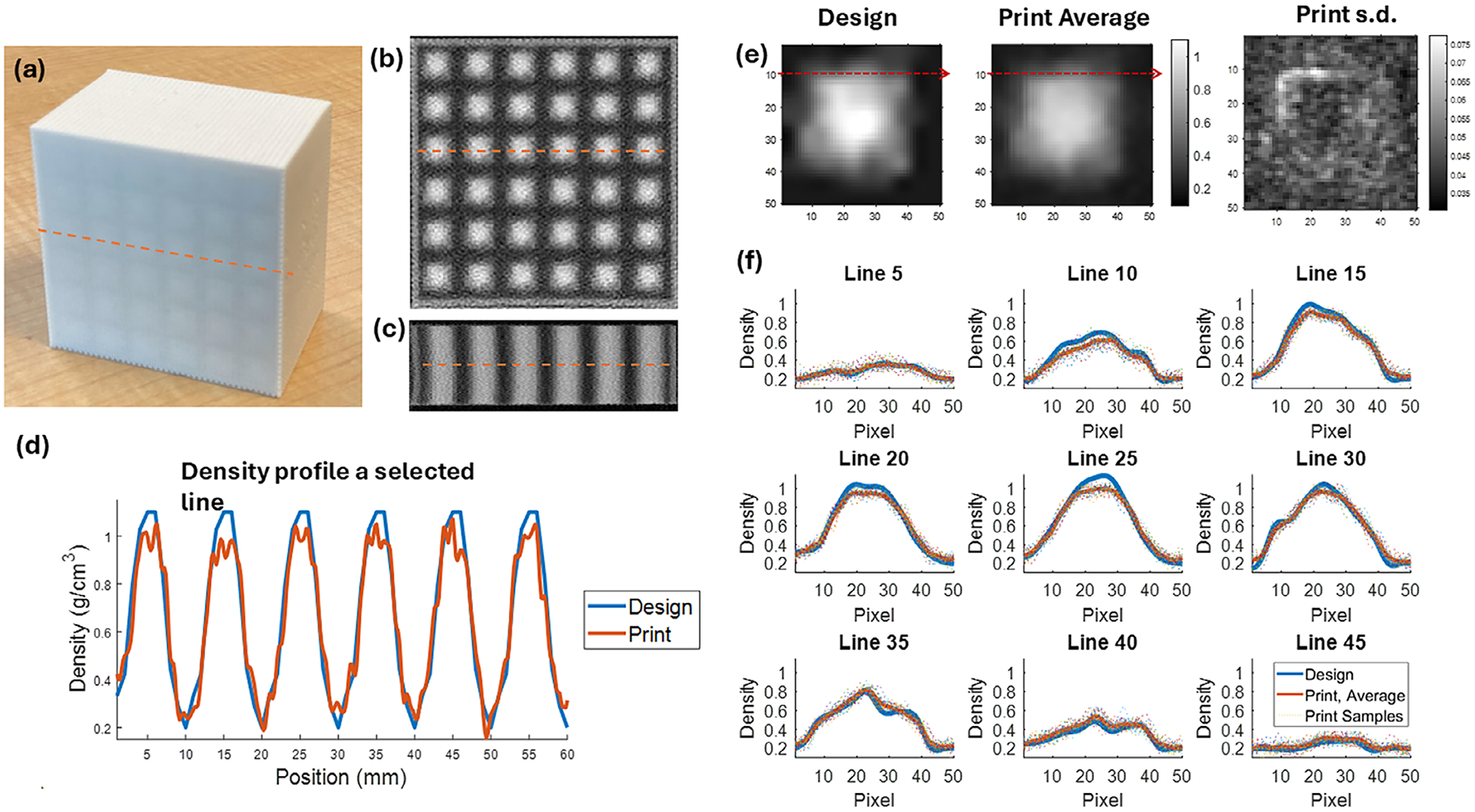
(a) 3D-printed 6 × 6 × 3.8 cm^3^ VDRMD with 3 cm SOBP. (b) Axial view of CT scan and (c) sagittal view of CT scan. (d) Density profile along the selected line across the device. Blue solid line is the designed phantom and red solid line is the printed device. (e) From left to right: the original designed density pattern resampled to 0.2 mm resolution; the average of density patterns sampled from the printed device; the standard deviation of sampled patterns. (f) The density profiles along every five lines in the 50 × 50 pixels patterns. Line 10 is shown as red dotted arrow in Figure (e). The blue solid line is the designed density profile, the red solid line is the printed density and averaged over multiple samples, and the dotted line is the printed densities of the samples.

A 3 cm SOBP VDRMD with dimensions 6 × 6 × 3.8 cm^3^ was 3D printed. A picture of the phantom is shown in Figure 4a and its CT images are shown in Figure 4b,c. Unlike conventional ridge filters, the VDRMD from PixelPrint is a solid block with varying densities in the transverse plane and repeated patterns in the longitudinal direction. The central region of the device shows good structural periodicity. However, at the periphery a minor distortion is observed (Figure 4c).

The printed VDRMD was scanned and reconstructed using 0.2 mm spacing in CT, to preserve spatial information. The optimized density distribution has 1 mm resolution but was upsampled to 0.2 mm for comparison with the printed density. The HU was then converted to density through the calibration acquired in Section III.1. The density profile along a line shown in Figure 4a–c is plotted in Figure 4d, with blue line showing the optimized VDRMD and red line being the printed device. The printed density profile reliably captures the intended variation, showing high fidelity at 1.0 mm. The low-density and intermediate-density regions are printed accurately, while density deviations are observed at the extremes of the high-density regions.

A close-up view of the densities and printing quality is presented in Figure 4e,f. The first figure in Figure 4e is a single block of the designed 10 × 10 mm^2^ density pattern interpolated from 1.0 mm resolution to 0.2 mm to match the scanned printed phantom, resulting in 50 × 50 pixels. The second figure in Figure 4e is the sampled average of density patterns from the printed device. The thirty-six 10 × 10 mm^2^ patterns in each slice are sampled by every five slices and extracted from the CT scan and averaged. Horizontal profiles are acquired every five pixels along the vertical direction. For example, Line 10 is shown in red dashed arrow, and the density profiles along the lines are plotted in Figure 4f between the design (blue solid) and average of print (red solid). The sampled patterns in the print are also shown as dotted in Figure 4f to show the variation of 3D print. The third image in Figure 4e is the standard deviation (s.d.) map of sampled density patterns.

On average, the spatial distribution of density is rendered well through PixelPrint. Comparing the design and averaged print, the shape and low-density regions are printed accurately. The high-density regions, however, show printed values that fall slightly below the expected levels. In the highest density region, the averaged printed density is 0.99 g/cm^3^ compared with 1.1 g/cm^3^ in design. In the samples, the mean difference of printed densities from the design is 0.049 g/cm^3^. If only focusing on the regions where the designed density is larger than 0.9 g/cm^3^, the mean difference is 0.083 g/cm^3^. From the repetitions across the printed phantom (dotted line in Figure 4f), a variation of printing is observed. The standard deviation of printing averaged over the 50 × 50 pixels is 0.046 g/cm^3^. As shown in Figure 4e, the low-dose and high-dose regions have similar s.d., and the regions with sharp density gradient have larger density variations.

### 3.4 | Experimental validation

**FIGURE 5:**
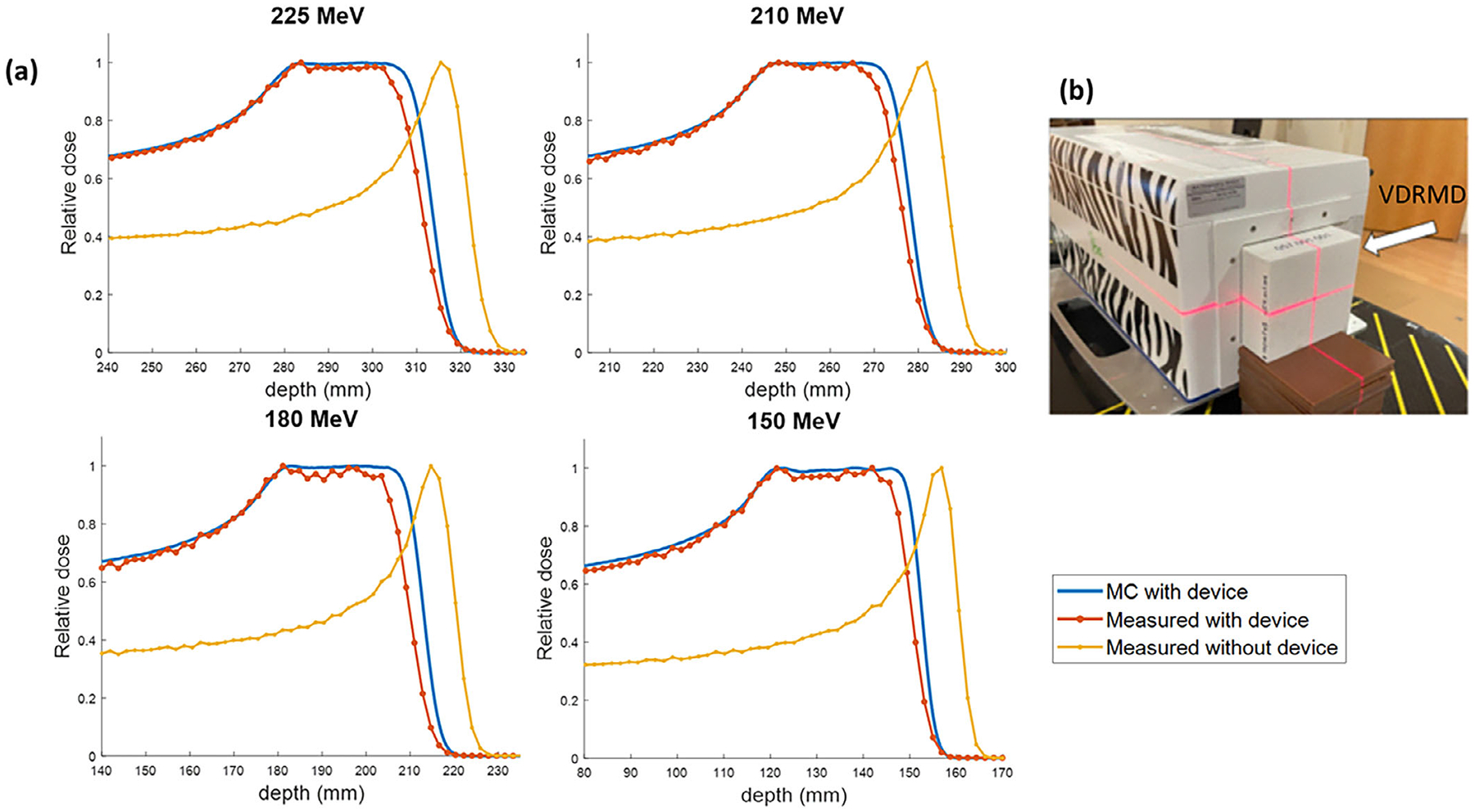
(a) The depth dose profiles under different proton beam energies for MC simulation (blue), MLIC measurement with the printed device (red), and MLIC measurement without the printed device in beam (yellow). (b) The experimental setup with MLIC. The printed VDRMD is positioned at the front window of the MLIC.

The feasibility of VDRMD was further demonstrated by experimental measurement with the MLIC under a monoenergetic PBS proton beam of 10 × 10 cm^2^ field size from 150 to 225 MeV. The setup is shown in Figure 5b with the device placed at the MLIC front window. The collected measurements are compared against MC simulation with the designed device, as shown in Figure 5a. The MLIC relative readings without the device in the beam are also plotted. Compared with the narrow pristine Bragg peak when no device is used, adding VDRMD to the beam path shows a modulation of the proton beam and a widening of SOBP. The distal edge of the depth dose curve with the device is shifted proximally from the pristine Bragg peak due to the nonzero minimum density used for modulation, which introduces a range offset. The SOBPs exhibit good flatness under different energies from 150 to 225 MeV. The width of the SOBP is slightly narrower than MC simulation, and the distal dose falloff is less sharp. The SOBP width is 29.7 mm for 225 MeV and 30.9 mm for 150 MeV, with a maximum difference of 1.6 mm across the tested energies. The distal dose falloff is 7.3 mm for 225 MeV and 5.2 mm for 150 MeV, which on average is 1.4 mm higher than MC simulation. The detailed values are listed in Table 2.

**FIGURE 6:**
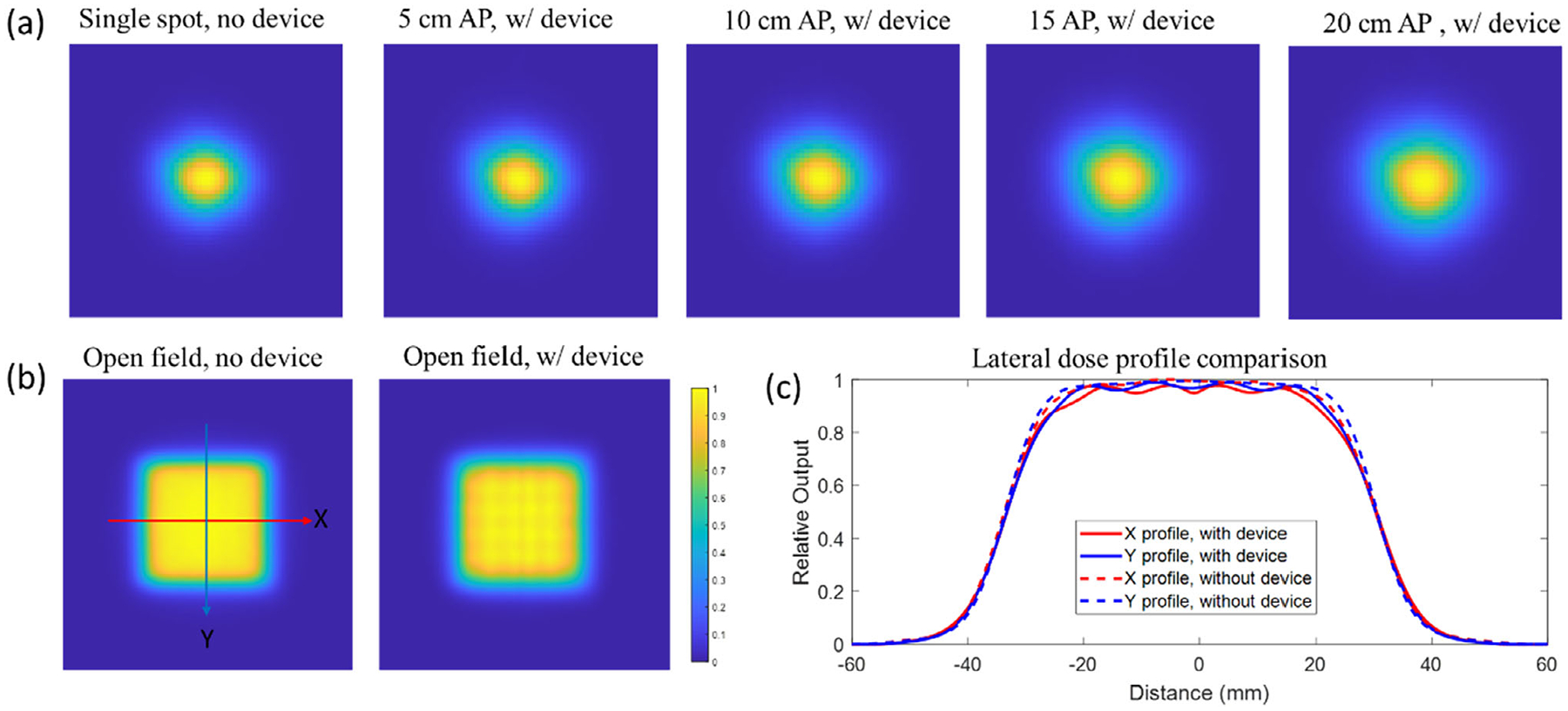
(a) Spot shape of a 180 MeV proton pencil beam without the modulator and with the modulator inserted, with an air gap (AP) ranging from 5 to 20 cm between the modulator and the Lynx surface. (b) Lateral dose profiles of a 5 × 5 cm^2^ 180 MeV proton field measured at a 19 cm depth (mid-SOBP plateau) with and without the device. (c) Extracted *X* and *Y* dose profiles from the selected line profile.

In addition to depth dose profile, the spot size and lateral dose profile were also measured and shown in Figure 6 and Table 3. Compared to the in-air spot size without device in beam, inserting the 3D printed modulator into the beam path increases the spot size in *X* and *Y* direction from 3.03 and 2.78 mm to 3.12 and 2.93 mm, respectively, with a 5 cm air gap. Increasing the air gaps enlarged the spot size. With a 20 cm air gap, the spot sizes were 3.84 and 3.68 mm in *X* and *Y* directions. For the lateral dose profile measured in the middle of the SOBP plateau, the modulator introduced a grid-like pattern with a peak-valley ratio of 0.96. Moreover, the lateral penumbra expanded from 9.7 and 8.6 mm (without the device) to 11.3 and 10.2 mm in the *X* and *Y* directions, respectively. Overall, while the modulator slightly increases the spot size and penumbra, it maintains a homogeneous lateral dose distribution.

## 4 | DISCUSSION

Cyclotron-based proton therapy systems such as the Proteus Plus system (IBA Proton systems, Belgium) are capable of delivering nozzle beam currents of up to several hundred nano-amperes^11^ corresponding to an instantaneous dose rate exceeding several hundred Gy/s when utilizing the highest proton energy of 227 MeV. The system’s efficiency drops significantly when utilizing the energy selection system. In addition to the time delay caused by energy switching, degrading the beam energy to 200 and 150 MeV results in the nozzle current dropping to 25% and 5% of peak value, respectively.^11^ FLASH conditions are thus only achievable on existing clinical systems utilizing the peak nozzle current without energy switching. This necessitates the use of ridge or energy filters in the beam path to create the different energy layers required to generate a spread-out BP.

Traditional 3D-printed uniform ridge filters exhibit multiple deficiencies, such as modulation flexibility, structure height limitations, printing fidelity, printing defects, and decreased robustness of filter performance when subjected to positional variations owing to sharp oblique edges relative to the beam direction. Utilizing PixelPrint technology, we explored a novel class of variable density filters that will potentially address the limitations of traditional ridge filter design, thereby enhancing the feasibility of FLASH particle therapy in clinical settings. The solid design of these filters, free from structural spikes, improves integrity and robustness. By shifting from height-based to density-based modulation, we can potentially achieve greater flexibility in energy modulation for particle therapy. In this work, we demonstrated that the 2D lateral variation in density distribution effectively generates a universal SOBP. Additionally, further modulation of density along the beam direction introduces additional degrees of freedom, which can be advantageous in patient-specific therapy planning. PixelPrint technology’s capability to achieve fine resolutions as precise as 0.1 mm holds potential for more accurate proton beam delivery.

An integrated framework has been developed to optimize and validate variable density devices that enable the generation of uniform SOBPs from pristine Bragg peaks. The optimization process directly generates a printable density map, capable of forming various SOBPs, which was rigorously validated through Monte Carlo simulations. The dose profiles derived from MC simulations demonstrated agreement with the analytically optimized SOBP profiles, confirming the feasibility of density profile optimization using analytical methods. This approach can be further extended to more complex applications, such as device optimization for patient-specific treatment plans. Although the distal edge of the dose distribution from MC simulations aligned well with analytical predictions, a slight enhancement of approximately 1% in the proximal dose region was observed, likely due to a change in scattering conditions introduced by the variable materials within the beam path.

Three simplifying assumptions were introduced in Section II.2 to reduce the complexity of the optimization problem and to facilitate an analytical formulation. Although these assumptions are commonly used in prior studies^13^ for traditional ridge filters, they may introduce deviations between the assumed analytical beam characteristics and those of a realistic clinical beam. Proton beams in our institution have a nominal source-axis distance (SAD) of 230 cm. For a 6 × 6 cm^2^ phantom, the beam divergence angle at the phantom’s edge is less than 1°, and even smaller along the central axis. The impact of this minimal divergence on the filter design is therefore subtle. Although beam fluence attenuation does not significantly affect the SOBP shape—or thus substantially alter the filter design—it must still be accurately modeled for dose calculation and output verification after the beam passes through the modulation device. It will be further investigated in future work.

The primary source of discrepancies between analytically derived SOBP profiles and those obtained from MC simulations is the scattering condition, which is not explicitly considered in the analytical calculation. To mitigate this issue, we introduced total variation regularization to promote piecewise density smoothness within the device, thereby reducing scatter-induced variations in the proton beam. Although such scatter cannot be entirely eliminated, the analytical results and MC simulations are largely consistent, with only minor differences observable at the proximal edge, as illustrated in Figure 3. In future applications, such as the development of patient-specific modulators, if deviations between analytical and MC results become pronounced, the MC-derived data can serve as a reference to refine and improve the device design. This iterative feedback loop will ensure that the optimized modulators more accurately reflect realistic beam conditions.

We successfully 3D printed the first variable density modulation device and demonstrated its feasibility of range modulation through experimental validation. With the variable density device placed in the beam, SOBP width around 3 cm is created across multiple proton energies from 150 to 225 MeV, with maximum variation of 1.6 mm across the energies. Excellent SOBP flatness is also observed in measurements. This is the first experimental demonstration of Bragg peak modulation using a 3D-printed variable density device.

The printed device exhibited overall excellent agreement between the designed and printed densities. Specifically, the shape and low-density regions closely matched the design specifications. However, high-density regions were printed with densities lower than expected. In the highest density regions, the average printed density was 0.11 g/cm^3^ lower than the designed 1.1 g/cm^3^, with a mean density difference of 0.083 g/cm^3^. Across the entire phantom, mean density difference between the design and the print was 0.049 g/cm^3^. The discrepancy in the high-density region may be due to the limitations of fused deposition modeling (FDM) 3D printing technology, for it is difficult to distribute 100% filament in the sample. Limitations in CT imaging could also lead to underestimation of actual high densities. Additionally, there were challenges in rapidly varying densities, such as printing high-gradient density profiles. Inconsistencies in the printing process were also observed, with a standard deviation of 0.046 g/cm^3^, the most significant variation occurring in regions with high density gradients. A minor distortion was present in the periphery of phantom, which is associated with printing speed. Lower printing speed can mitigate the geometrical distortion but reduce the printing efficiency, which is trade-off to be explored in future work.

Figure 5 and Table 2 show a difference in SOBP width between MC and measurements between 3 and 4.4 mm and a difference in distal falloff around 1.4 mm. The narrowing of SOBP width and the broadening of the distal dose fall-off observed in measurements compared to MC simulation primarily stems from the fact that MC simulations assume ideal density distributions, whereas actual 3D printing introduces deviations in printed densities (as shown in Figure 4). Although imperfections in 3D printing are undesirable, they are anticipated given current technological limitations, typically resulting in a narrower SOBP width and less sharp dose fall-off.

This issue is not unique to our devices; similar challenges have been observed with traditional 3D-printed ridge filters. For instance, Roddy et al.^20^ reported that static ridge filters (HEDGEHOGs) consistently exhibited significant deviations between film measurements and MC simulations, including dose inhomogeneity along the depth profile, reduced SOBP width, and less sharp distal fall-off. A reduction in SOBP width by more than 5 mm and an increase in dose fall-off on the order of centimeters are thus common across various 3D printing approaches for ridge filters. Kim et al.^10^ reported a distal falloff (d_d_10–d_d_90) of approximately 25 mm using a 3D-printed ridge filter designed for a 2.5 cm SOBP. In contrast, our proposed device achieves an SOBP width that agrees with MC predictions within 5 mm and exhibits a sharp dose fall-off of less than 7.5 mm.

In future applications, the MC simulation can be modified to take account into the artifacts and uncertainties introduced by 3D printing. This can be achieved by incorporating the scanned CT image of the printed device directly into the MC simulation to evaluate how printing imperfections affect the resulting dose distribution. The comparison between the simulated and measured SOBP profiles can then be used to identify areas of discrepancy, allowing for iterative adjustments to the density map or HU-to-density calibration to improve printing accuracy and achieve optimal dose agreement.

Meanwhile, for proton FLASH therapy utilizing ridge filter, after every ridge filter is manufactured, a comprehensive quality assurance (QA) for the device is required to fully validate the modulator before clinical use. Under the current technique, before using this proposed variable density range modulator for clinical use, we also recommend performing a comprehensive QA to every device. This includes CT imaging to verify the printed density distribution and structural integrity against the design, as well as depth-dose and lateral dose profile measurements using high-resolution detectors such as a multi-layer ionization chamber and film.

Although this study serves as a proof-of-concept to demonstrate the feasibility of using a variable density modulator, future work will focus on refining the design methods and improving PixelPrint technology for this specific task to achieve more accurate results. Alternative approaches to improve printing accuracy include limiting the use of very high densities in PixelPrint and creating smoother density distributions during optimization. Additional parameters, such as pixel resolution, pixel-wise smoothness, and pattern spacing, will also be explored to optimize printing precision.

Another factor influencing dose modulation is the spatial arrangement of densities. Under the assumptions described in Section II.2, the analytical approach produces an SOBP that is independent on this spatial distribution. Therefore, the densities resulting from the optimization depend on the initial input distribution. Without appropriate design, a random initialization can generate scattered, suboptimal density patterns that introduce excess scattering and fail to produce a stable SOBP, as shown in Figure 3.

To address this, we employed a cone-shaped density distribution as the initialization, mimicking the well-established pyramid-like shape of traditional ridge filters. This shape not only benefits from prior validations demonstrating feasible proton range modulation but also offers smooth density variations that enhance 3D-printing accuracy. Although other spatial configurations may also prove effective, this proof-of-concept work focuses on demonstrating the feasibility of variable-density modulation. The cone-shaped design has been validated by both MC simulations and experimental measurements. Future investigations will explore diverse spatial distributions and their effects, aiming to further refine and optimize device performance.

This innovative approach to fabricating variable density range filters introduces an entirely novel approach of optimizing the modulator design with various clinical applications. It offers enhanced structural integrity and modulation flexibility, potentially advancing FLASH therapy research and facilitating future clinical trials in existing proton therapy centers. Although the current study focuses on the development and validation of generic devices to achieve universal SOBPs, such designs are highly valuable for preclinical FLASH applications, including animal studies and physics validation, where fast and robust beam modulation is essential. Importantly, the generic modulator developed in this study also serves as the enabling foundation for future patient-specific implementations. Although a transversely uniform density pattern was used here to demonstrate feasibility to create a SOBP in a box phantom, the same optimization framework can be extended to generate transversely spatially varying density distributions tailored to transversely conform dose to the target within each patient’s unique anatomy and treatment plan. This approach would parallel, but greatly expand upon, the concept of traditional ridge filters that are shaped to match local tumor thickness and depth, offering finer resolution, greater modulation flexibility, and potentially improved robustness. In a variable density design, the density of the range filter would modulate transversely to pull back the Bragg peaks to match the local tumor thickness and depth of each pencil beam.

Achieving this will require several key future developments. First, patient-specific geometric modeling must be incorporated by integrating the patient’s CT data directly into the density optimization process proposed in this work. This will allow the device to account for variations in tumor size, shape, and location, as well as the proximity of critical organs. Second, beamline-specific design integration will be necessary, including compatibility with range shifters, collimators, and scanning spot arrangements to ensure seamless operation within clinical treatment rooms. Third, the optimization algorithm must be expanded to include robustness criteria and dose/dose-rate objectives, enabling simultaneous control of spatial dose conformity and ultra-high dose rate delivery required for FLASH effects. Finally, a dedicated QA workflow for each patient-specific modulator must be established, including verification of physical density distribution, dimensional accuracy, and delivered dose profiles.

In this way, future patient-specific devices could combine the ultra-fast field delivery advantage of a static range modulator with the individualized treatment quality expected in modern proton therapy. By incorporating patient-specific tailoring into the same fabrication and optimization pipeline demonstrated here, treatment plans could achieve high conformality while maintaining FLASH dose rates, thereby preserving the speed, handling ease, and simplicity of the current design while directly addressing clinical accuracy requirements. Thus, the present work provides both proof-of-concept for the manufacturing and optimization platform and a clear roadmap toward full clinical translation.

## 5 | CONCLUSION

A novel variable density range modulating device for proton therapy was developed. MC simulation and experimental validation demonstrated its feasibility to produce SOBP via variable density modulation. These devices have the potential to be designed and manufactured for patient specific particle therapy treatment plans.

## Figures and Tables

**Table 1:** Comparison of SOBP width (d_d_90-d_p_90) between optimization and MC simulation of the three designed devices.

Designed SOBP width parameter W (mm)	Optimization (mm)	MC simulation (mm)	Difference (Opt-MC) (mm)	Percentage difference (Opt-MC) (%)
30	34.4	33.1	1.3	3.8%
20	25.0	24.5	0.5	2.0%
10	15.7	14.8	0.9	5.7%

**Table 2:** Comparison of SOBP width (d_d_90-d_p_90) and distal dose falloff (d_d_20-d_d_80) between MC and measurements with the printed device.

Proton energy	SOBP width (mm)			Distal dose falloff (mm)		
	MC	Measur.	Difference	MC	Measur.	Difference
225 MeV	33.4	29.7	3.7	5.7	7.3	−1.5
210 MeV	33.7	30.8	3.0	5.6	6.9	−1.3
180 MeV	33.7	29.3	4.4	4.9	6.3	−1.4
150 MeV	34.2	30.9	3.2	3.8	5.2	−1.4

**Table 3:** Spot sizes (*σ*_X_ and *σ*_Y_) of a 180 MeV proton pencil beam measured in the *X* and *Y* directions under two conditions: (1) without a device in the beam path, and (2) with a device inserted. For the latter, measurements were taken with air gaps ranging from 5 to 20 cm between the modulator and the Lynx.

	*σ*_*X*_ (mm) Make	*σ*_*Y*_ (mm)
No device	3.03	2.78
5 cm air gap, w/ device	3.12	2.93
10 cm air gap, w/ device	3.30	3.09
15 cm air gap, w/ device	3.60	3.44
20 cm air gap, w/ device	3.84	3.68
